# Letter to the editor: “Mediation effect of healthy lifestyles on the association of socioeconomic status with mortality among US cancer survivors: a population-based cohort study”

**DOI:** 10.1097/JS9.0000000000003344

**Published:** 2025-09-10

**Authors:** Xiaoyan Shi, Peng Zhang

**Affiliations:** a Department of Hematology, The First Affiliated Hospital of Dalian Medical University, Dalian, People’s Republic of China; b Department of Urology, The First Affiliated Hospital of Dalian Medical University, Dalian, People’s Republic of China

*Dear Editor,*

We read with great interest the article “Mediation effect of healthy lifestyles on the association of socioeconomic status with mortality among US cancer survivors: a population-based cohort study.” This study focused on how healthy lifestyles mediated the impact of socioeconomic status (SES) on mortality among 2411 cancer survivors in the National Health and Nutrition Examination Survey (NHANES) 2000–2013, revealing that promoting healthy lifestyles could mitigate a substantial proportion of socioeconomic inequities in cancer-specific outcomes^[1]^. However, there are deeper reflections in this study. We have been assured that the article complies with TITAN Guidelines 2025-governing declaration and use of AI^[2]^.

First, although this study innovatively explored the mediating role of healthy lifestyles in the relationship between SES and mortality among US cancer survivors, the method used to construct a healthy lifestyle score has limitations. Data from Supplementary Table 3 demonstrates highly unequal contributions of individual lifestyle factors to mortality risk: no heavy alcohol drinking (hazard ratio [HR] = 1.15, 95% confidence interval [CI]: 0.78–1.68, *P* = 0.484) and healthy diet (HR = 0.88, 95% CI: 0.74–1.06, *P* = 0.185) were not statistically significant; never smoking showed only marginal significance (HR = 0.83, 95% CI: 0.68–1.02, *P* = 0.071); only physical activity demonstrated a consistently strong effect (HR = 0.57, 95% CI: 0.47–0.70, *P* < 0.001). However, the current study used a simple additive scoring method for the healthy lifestyle score, assigning one point for each healthy behavior, assuming that all behaviors have the same impact on health. To quantify the mediating effects of lifestyles more accurately, it is recommended to use an HR-weighted scoring method: use Cox regression to analyze the HR between each lifestyle factor and mortality separately, convert the HR into a weighting coefficient, and then calculate the weighted total score. This approach reflects the heterogeneous impact of different healthy behaviors, avoids bias caused by the “dominant effect of physical activity” and enhances the validity of mediation analysis.

Second, subgroup analyses revealed significant racial heterogeneity in the mediating effects of healthy lifestyles (Supplementary Table 6). In the non-White population, healthy lifestyles mediated only 7.2% (95% CI: 1.4–29.0; *P* = 0.071) of the effect of SES on cancer mortality, and 5.3% (95% CI: 1.3–19.1; *P* = 0.061) of the effect of SES on all-cause mortality. These mediation effects did not reach statistical significance or exceed the practical significance threshold (10%), providing insufficient evidence for a clinically meaningful mediating role of healthy lifestyles in the association between SES and mortality among non-White cancer survivors. This finding raises concerns regarding the generalizability of this study. On one hand, the authors’ conclusions may not be generalizable to the non-White population, as the mediating effects of healthy lifestyles on the association between SES and mortality exhibit racial heterogeneity. However, the assessment of SES may have cross-racial measurement biases, and the non-White population may not be homogeneous. Combined analysis, limited by insufficient samples, may mask critical heterogeneity^[3]^. Future studies should expand the sample size (e.g., by combining more cycles of the NHANES) and report the mediating effects of race. Consequently, while strategies promoting healthy lifestyles may effectively reduce SES-based health disparities in White cancer survivors, structural interventions are likely necessary to decrease these disparities in the non-White population.

There are several explanations that merit consideration. First, the limited sample size of non-White participants likely reduced the precision of the subgroup estimates. Second, structural barriers, including reduced access to healthy food, safe exercise environments, and culturally tailored health education, may constrain the adoption of healthy lifestyles in non-White communities. Third, SES indicators, such as education or income, may carry different meanings across racial groups due to systemic inequities, leading to measurement bias. To address these issues, future research should employ multigroup or Bayesian mediation models and adopt intersectional frameworks that account for the compounded effects of race, gender, and class. Bayesian approaches offer a flexible framework for handling small subgroup sizes and incorporating prior knowledge, which is especially valuable when examining marginalized populations.

In conclusion, this study, for the first time, employed a latent class analysis model to integrate income, occupation, education, and insurance into a composite SES indicator and combined this with a four-domain healthy lifestyle score to systematically quantify the mediating role of lifestyle in SES-associated mortality among US cancer survivors (8.7–28.7%), with cancer-specific deaths accounting for nearly one-third of this mediation. The results remained robust across sensitivity and subgroup analyses, providing directly applicable quantitative evidence for implementing dual-track medical-behavioral interventions to reduce socioeconomic disparities in cancer survival^[4]^.

## Data Availability

Not applicable. No datasets were generated or analyzed for this article.

## Research registration unique identifying number (UIN)

Not applicable. This work does not report a research study involving human participants and therefore does not require registration.
